# GE11-antigen-loaded hepatitis B virus core antigen virus-like particles efficiently bind to TNBC tumor

**DOI:** 10.3389/fonc.2023.1110751

**Published:** 2023-03-20

**Authors:** Long Zhang, Lin Tang, Yongsheng Jiang, Chenou Wang, Lijiang Huang, Ting Ding, Tinghong Zhang, Huaqiong Li, Longteng Xie

**Affiliations:** ^1^ Department of Infectious Diseases, The Affiliated Xiangshan Hospital of Wenzhou Medical University, Ningbo, Zhejiang, China; ^2^ Zhejiang Engineering Research Center for Tissue Repair Materials, Wenzhou Institute, University of Chinese Academy of Sciences, Wenzhou, Zhejiang, China; ^3^ School of Biomedical Engineering, Wenzhou Medical University, Wenzhou, Zhejiang, China

**Keywords:** virus-like particles, Ge11, triple-negative breast cancer, nanomedicine, drug delivery

## Abstract

**Purpose:**

This study aimed to explore the possibility of utilizing hepatitis B core protein (HBc) virus-like particles (VLPs) encapsulate doxorubicin (Dox) to reduce the adverse effect caused by its off-target and toxic side effect.

**Methods:**

Here, a triple-negative breast cancer (TNBC) tumor-targeting GE11-HBc VLP was constructed through genetic engineering. The GE11 peptide, a 12-amino-acid peptide targeting epidermal growth factor receptor (EGFR), was inserted into the surface protein loops of VLPs. The Dox was loaded into HBc VLPs by a thermal-triggered encapsulation strategy. The *in vitro* release, cytotoxicity, and cellular uptake of TNBC tumor-targeting GE11-HBc VLPs was then evaluated.

**Results:**

These VLPs possessed excellent stability, DOX loading efficiency, and preferentially released drug payload at high GSH levels. The insertion of GE11 targeting peptide caused improved cellular uptake and enhanced cell viability inhibitory in EGFR high-expressed TNBC cells.

**Conclusion:**

Together, these results highlight DOX-loaded, EGFR-targeted VLPs as a potentially useful therapeutic choice for EGFR-overexpressing TNBC.

## 1 Introduction

According to the statistical data from the WHO, cancer caused the leading death among all diseases in most countries and is an important barrier to increasing lifetime. In 2022, there will be approximately 4,820,000 and 2,370,000 new cancer cases and 3,210,000 and 640,000 cancer deaths in China and the USA, respectively (1). With diagnosed 2.3 million new cases in 2020, female breast cancer has exceeded lung cancer as the most commonly detected cancer (2). In the USA, breast cancer continues to be the most prevalent cancer with a number of annual cases, with 287,850 incidences in 2022 (3). The lack of ER, PR, and HER2 makes target therapy difficult to use in triple-negative breast cancer (TNBC), leaving cytotoxic chemotherapy as the main type of treatment (4, 5). Therefore, the development of a novel delivery system with enhanced target efficiency is still urgently needed (6–8).

Composed of natural biological building blocks, the VLPs exhibit great promise as an efficient targeted nanocarrier in medicine (9). Compared to synthetic nanoparticles, the VLPs as natural protein nanoparticles take the advantages of lower toxicity, easy biodegradation, and biocompatibility (10). The structure of VLPs is stable under a wide range of pH and temperature (11–13). Among a range of VLPs, HBc VLPs as the most commonly used model for basic medical research can be easily produced in all known expression systems (14). HBc VLPs take accurately defined composition, suitability for modification, capacity to self-assembly, and complete biocompatibility/biodegradability *in vivo (* 15, 16). HBc VLPs can maintain structural integrity after deletions, substitutions, or insertions in its two immunodominant loop regions (MIR) and C-terminal tail (17–22). Normally, exogenous targeting epitope was most commonly inserted into the MIR region (AA 78-82) of HBc by genetic engineering (23–25).

Actually, peptides represent a suitable alternative to monoclonal antibodies as active targeting agents (26).They are studied for drug delivery systems functionalization with the goal to achieve smart drug delivery systems. They have low immunogenic potential and show good penetration into solid tumor tissues. The GE11 peptide (YHWYGYTPQNVI) is reported to bind specifically to EGFR but is significantly minus mitogenic than EGF (27). It is much smaller than EGF, and it binds only to one EGFR region. Lots of studies suggested that the GE11 peptide is suitable for targeting EGFR-expressing tumors (28–30). GE11-targeted drug delivery systems include liposomes, polymer-based polyplexes, and filamentous plant viruses based or polymeric nanoparticles for diagnostic and anticancer and gene delivery applications (31). However, utilizing GE11-targted HBc VLP for TNBC therapy is rarely attempted.

In this study, we successfully obtained hybrid HBc VLPs, which presented a GE11 peptide. We examined HBc VLPs as drug delivery carriers in a model of TNBC cancer. Modified VLPs delivered DOX to EGFR-expressing cancer cells. Our results highlight DOX-loaded, EGFR-targeted VLPs as a potentially effective therapeutic option for EGFR-overexpressing TNBC.

## 2 Materials and methods

### 2.1 Preparation of GE11-HBc monomer

HBc sequence was synthesized by the company, and the GE11 peptide was inserted into the MIR region by SphI single enzyme digestion. The GE11-HBc was attached to a His tag at the end of the C-terminus to facilitate protein purification. The GE11-HBc sequence was cloned into the pET28a vector *via Xho*I and *Nco*I restriction enzyme sites. The plasmid pET28a-GE11-HBc was transformed into *Escherichia coli* BL21 (DE3) and cultured in Luria–Bertani (LB) medium at 37°C until the OD_600_ reached approximately 0.6–0.8; then, 0.5mM isopropyl-β-d-thiogalactoside (IPTG) was added to the culture, and cells were grown at 26°C overnight to induce protein expression. The protein was purified with Nickel affinity chromatography (GE Healthcare) as the product description described.

### 2.2 Preparation and purification of HBc VLPs

The purified protein was heated at 70°C for 20 min, then centrifuged at 10,000 rpm for 30 min to collect the supernatant. The supernatant was filtered through a 0.45-μm filter and subjected to ion exchange chromatography purification (GE Healthcare, Sepharose 4FF). The HBc VLPs were isolated by using sucrose density gradient centrifugation. Briefly, lower-density solutions were prepared by diluting with buffer (250 mM sucrose, 10 mM Tris–HCl, 1 mM EDTA, pH 7.4) to yield final sucrose concentrations (vol/vol) of 55%, 45%, 35%, 25%, and 15%. Crude protein was added to the top of the gradient and then centrifuged for 2 h at 35,000 rpm at 4°C. After centrifugation, the fractions were collected and analyzed using sodium dodecyl sulfate–polyacrylamide gel electrophoresis (SDS-PAGE) and transmission electron microscopy (TEM) images.

### 2.3 Preparation of DOX-loaded HBc VLPs

The DOX-loaded HBc VLPs were prepared by a thermal-triggered encapsulation strategy (32). The Hg particles were first treated with RNase for 3 h at 37°C to remove the RNA. DOX (0.2 mg/ml) was incubated with 0.2 mg/ml Hg at 50°C, 60°C, 70°C, and 80°C for 30, 60, 90, and 120 min. The OD_482_ of each group was measured after the removal of free DOX by desalting column.

To calculate the loading capacity, 1 mg/ml DOX was diluted to 0.05, 0.1, 0.15, 0.2, 0.25, and 0.5mg/ml, and the standard curve of Dox was obtained after the measurement of the absorbance at A 482 nm.

### 2.4 *In vitro* release

The *in vitro* release process of DOX in VLPs under GSH conditions was analyzed according to a previously reported method (33). In brief, 20 ml of HBc-DOX VLPs (containing 0.1 mg/ml DOX) was added to a dialysis tube (MWCO of 3.5 kDa). Drug release was carried out by incubating dialysis tubing containing HBc-DOX VLP in 1 L of various PBS stoste, which contained different concentrations of GSH (0, 0.02, 5, and 10 mM). Finally, 500 μl of the test solution was withdrawn at different time intervals (0, 12, 24, 36, 48, 60, and 72 h, followed by the addition of the same volume of fresh medium, and quantitative analysis by a UV–Vis spectroscopy at A 482 nm. The accumulative release (%) was acquired from the following equation:


$$\text{Accumulative release}(\%)=({\text{C}}_{\text{t}}\times {\text{V}}_{\text{t}}+{\text{Σ}}_{\text{i}}{\text{C}}_{\text{i}}\times {\text{V}}_{\text{i}})/{\text{W}}_{\text{total}}\times 100\%$$

where C_t_ and C_i_ are the concentration of the drug in the stoste at testing time point (t) and the concentration of the drug in the discarded medium at testing time points (i) before t, respectively; V_t_ is the volume of the stoste; V_i_ is the volume of discarded medium; and W_total_ is the total drug mass in VLPs.

### 2.5 Cytotoxicity assays

EGFR-positive MCF7 and MDA-MB-231 cells (1 × 10^4^ ) were seeded to each well of 96-well plates. The medium in each well was removed after 24 h of incubation. Then, HBc-GE11, HBc-GE11-DOX (equal to 0.2 mg/ml free DOX), and free DOX (0.2 mg/ml) were suspended in Dulbecco’s modified Eagle’s medium (DMEM) and added to cells. The cells were further cultured for 12, 24, 36, or 48 h before standard CCK-8 assay testing (Dojindo).

### 2.6 Cellular uptake

A total of 5×10^4^ MCF7, MDA-MB-231 or MDA-MB-453 cells (EGFR−) were seeded on a cover-slide system overnight. Subsequently, the cells were treated with PBS, HBc-GE11-DOX (equal to 1.086 mg/ml free DOX) and free DOX (1.086 mg/ml), respectively. After 0.5, 1, and 2 h incubation, the cells were washed three times with PBS and then fixed with cold 4% paraformaldehyde (PFA) for 1.5 h. The cell nucleus was stained with 1 µg/ml 4′,6-diamidino-2-phenylindole (DAPI) dye for 10 min. At last, a confocal laser scanning microscope was used to obtain the image.

### 2.7 Flow cytometry assays

For further quantification, MCF7 and MDA-MB-231 cells (1×10^5^ cells/well) were seeded in 12-well plates, respectively, in fresh medium containing HBc-GE11, HBc-GE11-DOX, and free DOX (calculated on DOX at a final concentration of 2 μmol/ml) for 2, 4, 6, 8, 10, and 12 h. After this, cells were collected and suspended in a cold PBS buffer, then analyzed by CytoFLEX flow cytometry (Beckman, USA).

## 3 Results and discussion

### 3.1 Generation and characterization of HBc-GE11

**Figure 1:**
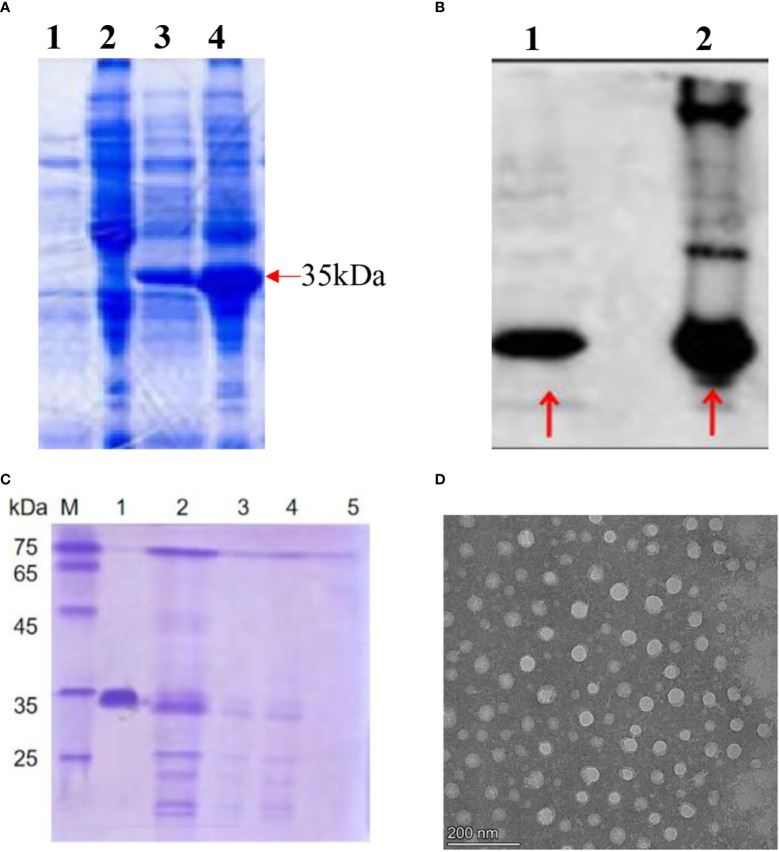
Production and morphology of the HBc-GE11 VLPs. **(A, B)** SDS-PAGE and Western blot analysis of HBc-GE11 protein expression. **(A)** Lines 1 and 2, bacterial lysate precipitation and supernatant before induction; lines 3 and 4, bacterial lysate precipitation and supernatant after induction. **(B)** Line 1, bacterial lysate precipitation after induction; line 2, bacterial lysate supernatant after induction. **(C)** Representative protein normalized SDS-PAGE of F1–F5. **(D)** Representative transmission electron micrographs of HBc-GE11 VLPs.

The HBc-GE11 protein was induced at 26°C overnight by 0.5 mM isopropyl β-d-1-thiogalactopyranoside (IPTG), and cells were lysed and purified by Nickel affinity chromatography (GE Healthcare). The expressed protein was subjected to SDS gel electrophoresis and Western blot analysis (**Figures 1A, B**). The HBc-GE11 monomer was observed as a ~35 kDa band. Interestingly, the multimeric complexes were also observed as higher-molecular-mass bands. The purified HBc-GE11 monomer was further purified by Sepharose 4FF ion-exchange chromatography and then subjected to sucrose density gradient centrifugation. The SDS-PAGE results suggest that VLPs mainly existed in a 15%–25% gradient fraction (**Figure 1C**, line2). Negative stain TEM analysis showed the existence of 40.481 ± 0.015 nm diameter vesicles (**Figure 1D**).

### 3.2 Generation of HBc-DOX VLPs

**Figure 2:**
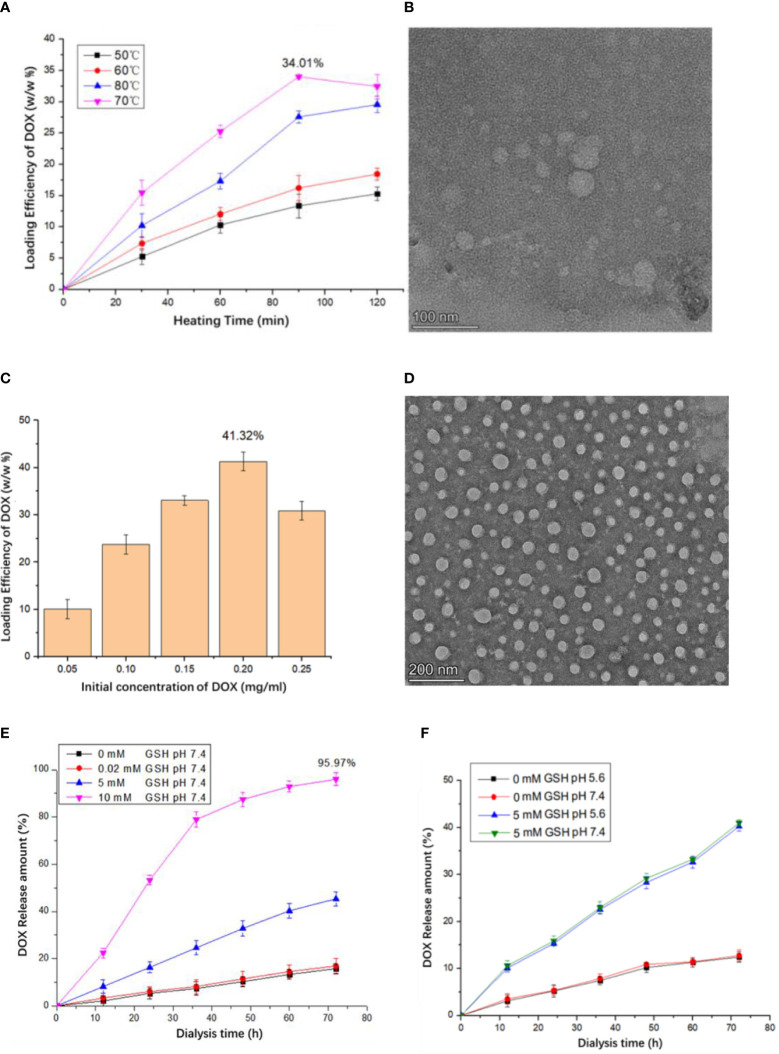
Production of the HBc-DOX VLPs and influence of different concentrations of GSH and Ph on drug release of HBc-DOX particles. **(A)** DOX loading at different heating temperatures and times. **(B)** TEM observation of HG particles heated at 70°C for 120 min. **(C)** Relationship between DOX concentration and loading rate. **(D)** HBc-DOX particles observed by TEM. **(E, F)** Influence of different concentrations of GSH **(E)** and Ph **(F)** on drug release of HBc-DOX particles.

It is reported that the thermal-triggered strategy can be applied to encapsulate the drug into VLPs in 10 min. By using thermally induced pore opening of the HBc capsid, 1,055 dye molecules could be encapsulated in each HBc VLP by simply mixing them at 60°C (34). Hence, a thermal-triggered encapsulation strategy was used for DOX encapsulation in this study. To find the optimal encapsulation condition, a fixed DOX and HBc concentration of 0.2 mg/ml was used. The initial temperature for encapsulation was set to 50°C. After being heated in a 70°C water bath for 90 min, 34.01% DOX was encapsulated into VLPs (**Figure 2A**). Compared to incubation at 60°C, a decreased encapsulation efficiency (EE) was observed, which was mainly due to the dissociation of the complete VLPs structure. It should be noted that some of HBc VLPs could not keep a complete structure after being heated 120 min at 70°C (**Figure 2B**). The optimal DOX encapsulation concentration was confirmed by incubating various concentrations of DOX (0.05, 0.1, 0.15, 0.2, and 0.25 mg/ml) with fixed HBc VLP (0.2 mg/ml) at 70°C for 90 min. The highest DOX loading capacity was observed when incubating 0.2 mg/ml DOX with 0.2 mg/ml HBc VLPs (**Figure 2C**). TEM result showed that DOX-loaded VLPs has a similar size to unloaded one (**Figure 2D**).

### 3.3 Release of DOX from VLPs under high GSH condition

It is well known that lots of disulfide bonds on the surface of HBc VLPs and high concentration of GSH in the tumor can reduce disulfide bonds and destroy HBc VLP structure. The concentration of GSH in the tumor site is up to 10 mM but only 0.02 mM in normal tissues (35). Thus, when HBc VLP particles enter the tumor cell, the high concentration of GSH will destroy the structure of HBc VLPs to release internal drugs. It can be seen from **Figure 2E** that when the GSH concentration is 0 and 0.02 mM (normal tissue cell concentration), the release of DOX in HBc-GE11-DOX is very small. When the GSH concentration is 5 mM, the release amount can reach 40.5% in 72 h, while 10 mM GSH treatment results in 95.97% drug release in 72 h. pH-dependent drug release is mostly used in anti-tumor drug design. To confirm whether pH influences VLPs drug release, HBc-DOX VLPs were exposed to different pH buffers under the same GSH concentration. There is no significant difference in the drug release of HBc-DOX under different pH conditions with the same GSH concentration, which indicates that HBc VLPs are not sensitive to pH (**Figure 2F**).

### 3.4 Cellular uptake of HBc-GE11-DOX VLPs

**Figure 3:**
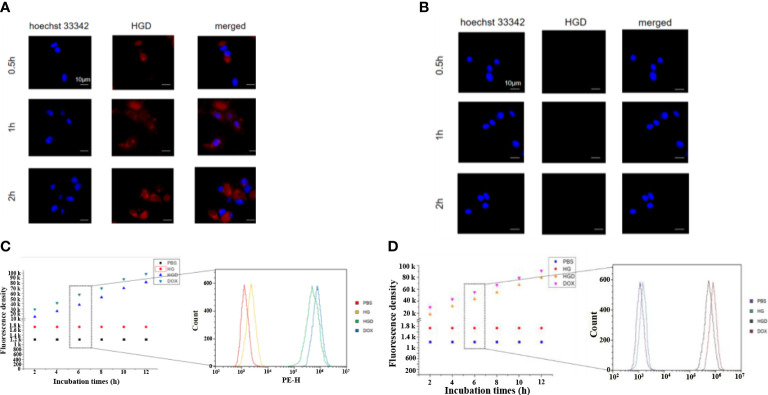
CLSM images after feeding free DOX and HBc-GE11-DOX to MDA-MB-231 **(A)** and MDA-MB-453 cells **(B)** at different time intervals. Red, DOX; blue, DAPI for cell nucleus. The scale bar is 10 μm. **(C, D)** Flow cytometry of MCF7 **(C)** and MDA-MB-231 cells **(D)** by the cellular uptake assay after feeding HBc VLPs, HBc-GE11-DOX, or free DOX for different times.

The cellular uptake of HBc-GE11-DOX was first assessed by confocal laser scanning microscope. The breast cancer cell lines (MDA-MB-231 and MDA-MB-453) were treated with free DOX and HBc-GE11-DOX, and images were obtained at different time intervals. The free DOX and HBc-GE11-DOX entered the MDA-MB-231 cell line very fast. Even at a feeding time as short as 0.5 h, the accumulation of DOX in cells was clearly noted for both DOX and HBc-GE11-DOX (**Figure 3A**). As the feeding time extended (from 0.5 to 2 h), more DOX accumulated in the cells for both HBc-GE11-DOX and DOX groups. Interestingly, at the same time intervals, the DOX cannot be found in HBc-GE11-DOX but not in free DOX-treated MDA-MB-453 cell, which is an EGFR-negative cell line (**Figure 3B**). We also used the FCM to evaluate the cellular uptake of HBc-GE11-DOX nanoparticles in both MCF7 (**Figure 3C**) and MDAMB-231 cells (**Figure 3D**). As shown in **Figures 3C, D**, all the cells are DOX-positive, suggesting that HBc-GE11-DOX and free DOX can easily be uptake by tumor cells.

### 3.5 Effect of HBc-GE11-DOX on killing breast cancer cells *in vitro*

**Figure 4:**
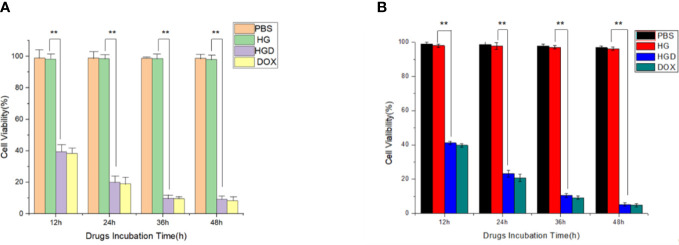
Viabilities of MCF7 **(A)** and MDA-MB-231 **(B)** after treating with 0.2 mg/ml of free DOX, HBc-GE11-DOX (equal to 0.2 mg/ml free DOX), or pure HBc VLPs for 12, 24, 36, and 48 h. **p < 0.01.

The cytotoxicity of HBc VLPs was valued first. No significant inhibition effect was observed on cellular viability in two different cancer cell lines with indicated HBc VLPs concentration and incubation times from 12 to 48 h (**Figure 4**). It is suggested that HBc VLPs have no or minimal cytotoxicity to the breast cancer cell lines. Subsequently, the HBc-GE11-DOX was used to treat two EGFR+ cells. The cytotoxicity of the HBc-GE11- DOX to two breast cancer cell lines was evaluated by CCK-8 kits, under various intervals of treatment times (12−48 h) (**Figures 4A, B**). PBS and pure VLPs groups were set up as the control, and no obvious cytotoxicity was observed, while notable cytotoxicity was observed for both free DOX and HBc-GE11-DOX groups. For both cell lines treated with different coincubation times, the cellular viability decreased with the increased incubation time (**Figure 4**). It should be pointed out that free DOX-treated cells exhibited lower cell viability, although no significant difference was observed.

## 4 Conclusion

Here, we constructed hybrid HBc-GE11 VLPs, which presented GE11 peptide to target EGFR+ breast cancer. We examined HBc VLPs as drug delivery carriers in a model of TNBC cancer. Modified VLPs delivered DOX to EGFR-expressing cancer tissues and exhibited a GSH-dependent drug release. Our results highlight DOX-loaded, EGFR-targeted VLPs as a potentially effective therapeutic option for EGFR-overexpressing TNBC.

## Data availability statement

The original contributions presented in the study are included in the article/supplementary material. Further inquiries can be directed to the corresponding authors.

## Author contributions

LZ and HL conceived and designed the project. LZ, TZ and HL prepared the manuscript. LT carried out the experiments and analyzed the data. CW did the materials characterization. YJ, HL and TD analyzed the data. LX and HL provided funding for this project. LZ and HL cowrote the manuscript. All authors contributed to the article and approved the submitted version.
